# Letermovir prophylaxis and early CMV DNAemia after allogeneic hematopoietic stem cell transplantation: a real‑world study from China

**DOI:** 10.1038/s41598-026-50851-1

**Published:** 2026-04-29

**Authors:** Juan Ren, Xiaoning Wang, Huachao Zhu, Pengcheng He

**Affiliations:** Department of Haematology, The First Affiliated Hospital of Xi’an Jiaotong University, 277 West Yanta Road, Xi’an, Shaanxi China

**Keywords:** Letermovir, Allogeneic hematopoietic stem cell transplantation, Cytomegalovirus, Prophylaxis, Diseases, Medical research, Microbiology

## Abstract

Letermovir is a cytomegalovirus (CMV) prophylactic agent that is not associated with clinically significant myelosuppression. However, real-world data regarding its clinical use in Chinese allogeneic hematopoietic stem cell transplantation (allo-HSCT) remain limited. This study aimed to assess CMV DNAemia during letermovir prophylaxis and its associations with other viral infections and clinical outcomes. We conducted a single-arm retrospective study of consecutive allo-HSCT recipients treated between July 2022 and December 2023 at the First Affiliated Hospital of Xi’an Jiaotong University. Patients who received letermovir within 28 days post-transplant were analyzed. The primary endpoint was the 100-day cumulative incidence of CMV DNAemia. Secondary endpoints included CMV end‑organ disease, acute graft‑versus‑host disease (aGVHD), other viral infections, and survival outcomes. Among 74 allo-HSCT recipients receiving letermovir prophylaxis, the 100‑day cumulative incidence of CMV DNAemia was 18.9% (95% CI, 10–28%), which remained similar after exclusion of CMV serostatus donor‑negative/recipient‑positive(D-/R+) cases (19.4%). The 100‑day cumulative incidence of aGVHD was 17.6%. In competing‑risk analyses treating death as a competing event, time‑dependent associations were observed between CMV DNAemia and aGVHD. Lower letermovir exposure (*p* = 0.036), delayed initiation of prophylaxis(*p* = 0.041), and use of high‑dose corticosteroids (*p* = 0.020) were more frequently observed among patients with breakthrough CMV DNAemia. This real‑world study describes the incidence and clinical characteristics of early CMV DNAemia in allo‑HSCT recipients receiving letermovir prophylaxis. Lower letermovir exposure and the use of high‑dose corticosteroids were more commonly observed among patients with breakthrough CMV DNAemia, highlighting clinical factors that warrant attention in this setting.

**Supplementary Information:**

The online version contains supplementary material available at 10.1038/s41598-026-50851-1.

## Introduction

Allogeneic hematopoietic stem cell transplantation (allo-HSCT) is widely used in the treatment of malignant hematologic diseases and is frequently complicated by cytomegalovirus (CMV) reactivation after transplantation. CMV DNAemia occurs in a substantial proportion of allo-HSCT recipients and has been associated with adverse post‑transplant outcomes, including higher non‑relapse mortality ^1–6^.

Letermovir, a non-nucleoside antiviral agent targeting CMV, is approved for prophylaxis in CMV IgG–positive allo-HSCT recipients ^7^ Evidence from randomized trials and meta-analyses indicates that letermovir has demonstrated a lower incidence of CMV infection compared with placebo (RR 0.19, 95% CI 0.13–0.29) ^8^.

In China, haploidentical HSCT is predominantly performed using the “Beijing Protocol,” which incorporates cyclosporine A (CsA), methotrexate (MTX), and mycophenolate mofetil (MMF) for graft-versus-host disease (GVHD) prophylaxis. All patients included in the present study underwent antithymocyte globulin (ATG)‑based haploidentical HSCT according to the Beijing Protocol. Cyclophosphamide was administered as part of the conditioning regimen, and post‑transplant cyclophosphamide (PTCy) was not used. While effective for GVHD prevention, this immunosuppressive strategy may increase the risk of CMV reactivation. Letermovir entered routine clinical use in China in 2022 ^9^, and a phase 3 study reported clinically significant CMV infection (csCMVi) in 32.7% of Chinese CMV-seropositive HSCT recipients, with no CMV end-organ disease observed within 24 weeks after transplantation ^10^.

However, factors associated with breakthrough CMV DNAemia during letermovir prophylaxis remain incompletely understood. Although antiviral resistance has been suggested, resistance-associated mutations are uncommon ^11^, leaving the roles of letermovir exposure and other transplant-related factors uncertain. In addition, real-world studies have reported changes in the patterns of other viral infections during letermovir prophylaxis ^12,13^.

Accordingly, this study aimed to describe the incidence and clinical features of CMV DNAemia and CMV end-organ disease, and to explore clinical and treatment‑related characteristics observed in patients with breakthrough CMV DNAemia.

## Methods

### Study design

This single-center, retrospective study included consecutive allo‑HSCT recipients treated at the First Affiliated Hospital of Xi’an Jiaotong University. This study was approved by the Ethics Committee of the First Affiliated Hospital of Xi’an Jiaotong University (No.XJTU1AF2023LSK-376) and was registered with the Chinese Clinical Trial Registry (ChiCTR2500100795).

### Objective and endpoints

The primary endpoint was the cumulative incidence of CMV DNAemia within 100 days post‑transplant. Exploratory analyses were performed to evaluate the temporal association between CMV DNAemia and the occurrence of aGVHD, as well as to describe clinical and treatment‑related characteristics observed in patients with breakthrough CMV DNAemia. Secondary endpoints included CMV end‑organ disease, other viral infections, and survival outcomes.

### Patients

Letermovir prophylaxis was implemented according to institutional practice, taking into account the estimated risk of CMV DNAemia ^14^ and treatment accessibility. All allo-HSCT patients were screened from July 2022 to December 2023. Patients were included if they met the following criteria: (1) receipt of first allo-HSCT; (2) CMV IgG seropositivity without CMV DNAemia pre-transplant; (3) initiation of letermovir prophylaxis within 28 days post-transplant; (4) scheduled CMV viral load monitoring at least weekly during the first 100 days post-transplant (≥ 10 assessments) and biweekly from day 100 to day 200 (≥ 5 assessments); and (5) provision of documented informed consent or assent if needed.

Letermovir (MSD, China) was administered orally at a dosage of 480 mg/day (or 240 mg/day for patients receiving cyclosporine). Acyclovir was administered post-transplant to prevent herpesvirus infections.

Diagnosis, grading, and treatment were conducted in accordance with guidelines ^15,16^. Underlying hematologic diseases were classified according to the World Health Organization classification of hematopoietic and lymphoid neoplasms (5th edition) ^9^. Pre-transplant disease risk stratification incorporated genetic risk at initial diagnosis based on the 2024 European LeukemiaNet guidelines and clinical manifestations ^17^. Conditioning regimen, graft source, and GVHD prophylaxis strategies were individualized according to the underlying disease and pre-transplant clinical status. The majority of patients received ATG‑based haploidentical HSCT following the Beijing Protocol, with cyclophosphamide used during conditioning (Supplementary Table 1). Among the cohort, one patient received a lower ATG dose (5 mg/kg) because of an older parous donor, while two patients without aplastic anemia were transplanted without ATG.

### Data collection

Trough plasma concentrations of letermovir(C_min_) were defined as the concentrations measured immediately prior to the administration of the subsequent dose, starting on the third day of treatment. Plasma samples were collected at least once weekly. C_min_ was measured using a validated high-performance liquid chromatography–tandem mass spectrometry (HPLC–MS/MS) method with a 3000 HPLC system (Thermo Fisher Scientific) coupled to a TSQ Vantage triple quadrupole mass spectrometer. For patients with multiple letermovir (C_min_) measurements, analyses were conducted at the patient level; specifically, each patient contributed a single summary value (e.g., the median trough concentration during prophylaxis) to avoid treating repeated measurements as independent observations.

Breakthrough CMV DNAemia was defined as CMV quantitative polymerase chain reaction (qPCR) ≥ 500 copies/mL (780 IU/mL according to the WHO standard) ^18^. Upon detection of CMV DNAemia, preemptive therapy was initiated. Intravenous ganciclovir (5 mg/kg every 12 h) was used as first-line therapy. If CMV viral load did not decrease or continued to rise after 10–14 days, switching to foscarnet (90–120 mg/kg/day, divided into two doses) was considered. Simultaneously, the intensity of immunosuppressive therapy was adjusted as needed and combined with other agents or immunoglobulin. Treatment continued until CMV DNA was negative in two consecutive tests. Following discontinuation of letermovir prophylaxis, CMV DNAemia was monitored by qPCR throughout follow-up, and preemptive therapy was initiated when predefined institutional criteria were met.

EBV DNAemia was monitored using a surveillance strategy similar to that for CMV, and managed according to institutional practice and established guidelines ^4,19,20^. BK polyomavirus–associated hemorrhagic cystitis (BKPyV‑HC) was defined base on standard clinical and virological criteria and managed with supportive care and adjustment of immunosuppressive therapy ^20^.

All patients were followed until March 1, 2024. Hematopoietic recovery was monitored, with neutrophil engraftment defined as the first of three consecutive days with an absolute neutrophil count ≥ 0.5 × 10⁹/L, and platelet engraftment defined as the first of seven consecutive days with platelet counts ≥ 20 × 10⁹/L without transfusion support. The onset of acute GVHD was defined as the first day of clinical diagnosis requiring initiation of methylprednisolone therapy. Overall survival (OS) was defined as the time from transplantation to death from any cause or last follow-up. Progression-free survival (PFS) was defined as the time from transplantation to relapse, death, or last follow-up.

### Statistical analysis

Statistical analyses and figures were performed using SPSS, R, and GraphPad Prism. Categorical variables were analyzed using the chi-square test, the corrected chi-square test, or Fisher’s exact test, as appropriate. Continuous variables with normal distributions were summarized as mean ± standard deviations (SDs), while variables with skewed distributions were summarized as median with interquartile ranges (IQRs). Exploratory subgroup analyses were performed using the Student’s *t*-test or the Mann-Whitney *U* test when appropriate. A sensitivity analysis excluding D−/R + CMV serostatus cases was performed to assess the robustness of the primary findings.

Time-to-event analyses were restricted to events occurring within the first 100 days after transplantation. The event of interest was the occurrence of CMV DNAemia or aGVHD, as specified in each model. Given the presence of a competing risk (death), cumulative incidence functions were estimated and visualized, and competing-risk regression was performed in an exploratory framework using the Fine–Gray subdistribution hazard model. Death was considered the only competing event, as other post-transplant events did not preclude the occurrence of CMV DNAemia or aGVHD within the study window. The proportional subdistribution hazards assumption was formally tested and met (*P* > 0.05 for all models). Subdistribution hazard ratios (sHRs) with 95% confidence intervals were reported, and all results were interpreted as associations rather than causal effects.

## Results

### Baseline characteristics

A total of 74 allo-HSCT patients were enrolled between July 2022 and December 2023. Detailed participant characteristics are presented in Table 1.

**Table 1:** Characteristics at baseline of 74 patients.

Characteristic (Total, *N*)	(*N* = 74)
Age, years, median (range)	35.5(10ཞ64)
Sex: Female/Male	30/44
Primary reason for hematopoietic-cell transplantation	
AML	32(43.24%)
Moderate risk	16(50.00%)
High risk	16(50.00%)
ALL	19(25.67%)
B-ALL	13(68.42%)
T-ALL	6(31.58%)
MDS	12(16.22%)
AA	10(13.51%)
SAA	7(70.00%)
NSAA	3(30.00%)
other	1(1.35%)
Pre-transplant status	AL(51)/MDS(11)
CR(CR1/CR2/CR3)	46(74.19%) (39/6/1)
MRD positive	22(47.83%)
NR	16(25.81%)
HLA matching and donor type	
Haploidentical related donor	62(83.78%)
Matched related	6(8.11%)
Matched unrelated	6(8.11%)
Gender of donor and recipient (consistent/inconsistent)	40(54.05%)/34(45.95%)
CMV recipient seropositive	
recipient	74(100%)
donor	72(97.30%)
Stem cell source	
Peripheral blood	57(77.03%)
Peripheral blood+ Bone marrow	5(6.76%)
Peripheral blood + Cord blood	12(16.22%)
GVHD prophylaxis	
Cyclophosphamide/Mycophenolate Mofetil/MTX	72(97.30%)
Cyclosporine/Mycophenolate Mofetil/MTX / Basiliximab	2(2.70%)
Anti-thymocyte globulin	
0	2(2.70%)
5 mg/kg	1(1.35%)
10 mg/kg	71(95.95%)

1. Other diagnoses refer to one case of chronic myelomonocytic leukemia

2. In the “Pre‑transplant status” row, MDS includes one case of hypoplastic myelodysplastic syndrome (MDS-h) , which was not included in the pre-transplant diseas status assessment.

3. Minimal residual disease (MRD) assessment included two methods: multiparameter flow cytometry (MFC) and real-time quantitative polymerase chain reaction (RQ-PCR). For cases with well-defined molecular markers, RQ-PCR results were used as the reference standard. Otherwise, MFC results were considered.

AML: Acute myeloid leukemia; B-ALL: B-cell acute lymphoblastic leukemia; T-ALL: T cell acute lymphoblastic leukemia; MDS: myelodysplastic syndrome; AA: Aplastic anemia; SAA: severe aplastic anemia; NSAA: non-severe aplastic anemia; CR: Complete response; NR: non-remission; HLA: human leukocyte antigen; CMV: Cytomegalovirus; GVHD: Graft versus host disease; MTX: Methotrexate.

### Incidence of breakthrough CMV DNAemia and CMV end-organ disease

The cumulative incidence of breakthrough CMV DNAemia was 18.9% (95% CI,10.0 - 28.0%; 14/74). Exclusion of the two D−/R+ cases resulted in a 100‑day cumulative incidence breakthrough CMV DNAemia was 19.4% (14/72). The median peak viral load was 1,020.50 copies/mL (IQR, 3,591.25 copies/mL). The median time to onset of CMV DNAemia was 44 days post-transplant (IQR, 21 days; range, 20–83 days). The mean duration of preemptive treatment for CMV DNAemia was 12 ± 5 days. The mean duration of letermovir prophylaxis was 84 days. Thrity patients did not complete the planned 100-day prophylaxis, primarily due to financial constraints, whereas 34 patients extended prophylaxis beyond 100 days because of persistent high-risk clinical factors, with a mean extended duration of 116.59 ± 17.94 days (range, 101–174 days).

**Fig. 1:**
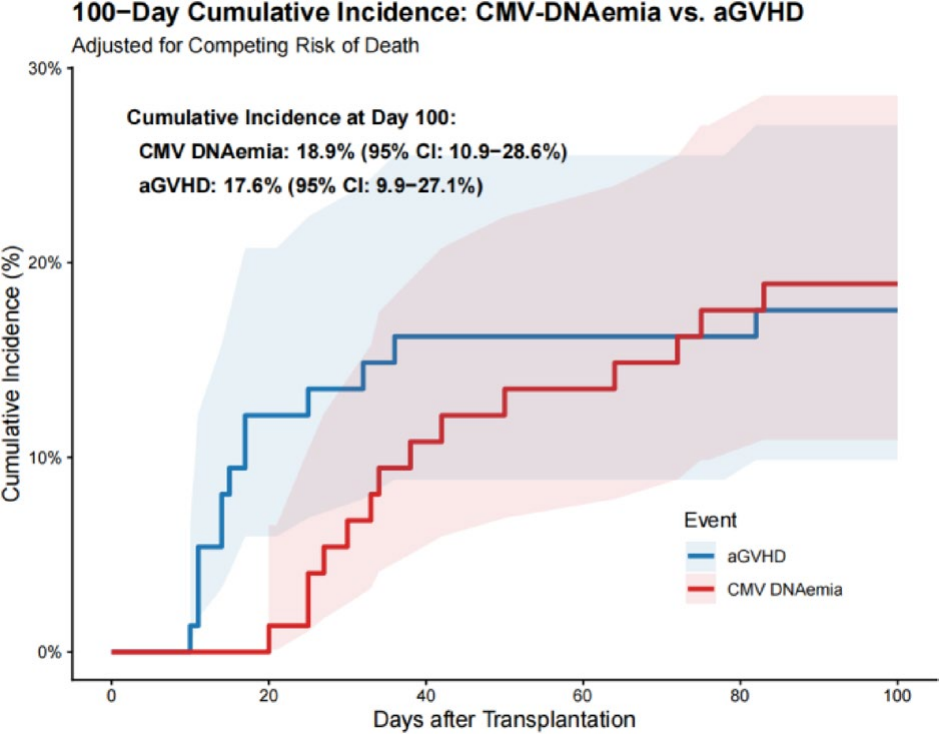
100-day cumulative incidence of breakthrough CMV DNAemia vs. aGVHD post-transplant adjusted for competing risk of death.

The cumulative incidence of aGVHD was 17.6% (95% CI, 9.9 - 27.1%,13/74). Among patients who developed aGVHD, CMV DNAemia was observed in 38.4% (5/13). Within the first 100 days after transplantation, aGVHD occurred earlier than CMV DNAemia. The median time to onset of aGVHD was 15 days, Among the five patients who developed both events, aGVHD consistently preceded the occurrence of breakthrough CMV DNAemia. (Fig. 1)

Reciprocal Fine–Gray competing-risk regression models were used to assess the temporal associations between breakthrough CMV DNAemia and aGVHD, with death treated as the competing event. Two separate models were constructed in a reciprocal manner: one evaluating aGVHD as a time-to-event outcome, with prior breakthrough CMV DNAemia as the exposure, and the other evaluating breakthrough CMV DNAemia as the outcome with prior aGVHD as the exposure. Breakthrough CMV DNAemia was associated with aGVHD (sHR, 3.09; *P* = 0.042), and aGVHD was associated with breakthrough CMV DNAemia (sHR, 3.05; *P* = 0.042). These findings reflect time-dependent associations rather than causal relationships.Between days 100 and 200 post-transplant, the incidence of breakthrough CMV DNAemia was 1.35%. All patients who developed breakthrough CMV DNAemia received preemptive antiviral therapy. Among the 14 patients, 8 continued letermovir as secondary prophylaxis following completion of preemptive treatment. All patients receiving second prophylaxis had undergone haploidentical transplantation with ATG, and five required high‑dose corticosteroid therapy for aGVHD. The duration of secondary prophylaxis was 52 ± 28 days (range, 10–95 days), continuing until day 100 post-transplant or, if the patient’s clinical condition permitted, until the end of follow-up. No recurrent breakthrough CMV DNAemia was observed during secondary prophylaxis in this subgroup.

One patient developed CMV pneumonia and was treated with IV ganciclovir combined with immunoglobulin. After two weeks of treatment, CMV DNA was undetectable in two consecutive tests, and the patient remained alive at the follow-up.

After discontinuation of letermovir prophylaxis and throughout follow-up, CMV reactivation was observed in 8.45% (6/71) of patients. Three patients died from transplant-related causes before CMV reactivation and were therefore excluded from the risk set. The mean time to CMV reactivation 63.62 ± 56.40 days after cessation (range, 6–164 days).

### Factors affecting breakthrough CMV DNAemia

**Fig. 2:**
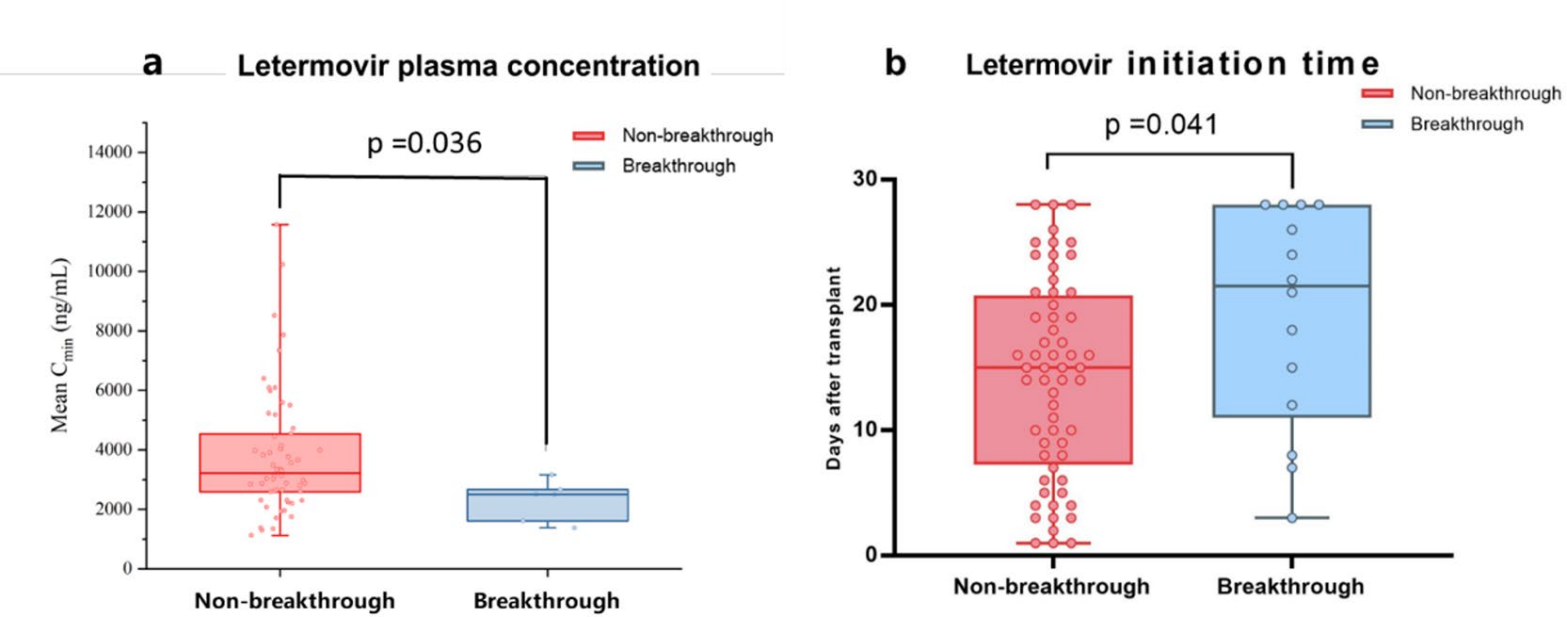
Letermovir use and descriptive analyses of breakthrough CMV DNAemia. (**a**) Patient‑level time to first breakthrough CMV DNAemia during letermovir prophylaxis. (**b**) Distribution of letermovir initiation timing after transplantation (median, IQR).

Letermovir Cmin monitoring was performed in 64 patients, including 6 patients with breakthrough CMV DNAemia (41 samples) and 58 patients without breakthrough CMV DNAemia (591 samples). The mean letermovir C_min_ was lower in patients with breakthrough CMV DNAemia compared with those without breakthrough (2,303.06 ± 677.27 ng/mL vs. 3,831.43 ± 2,126.40 ng/mL, *P* = 0.036). Patient-level analyses of letermovir exposure are shown (Fig. 2a).

The mean time to initiation of letermovir prophylaxis was 15 days post-transplant. Patients who developed breakthrough CMV DNAemia initiated letermovir significantly later than those without breakthrough (19 ± 9 days vs. 14 ± 8 days, *P* = 0.041) (Fig. 2b).

Additional clinical variables were evaluated based on previously published literature ^21,22^, and the results are summarized in Table 2. In univariate analyses, the following variables were examined for associations with breakthrough CMV DNAemia: patient age and sex, donor age and sex, patient-donor sex matching, HLA compatibility, diagnosis of AA, and use of high‑dose corticosteroids (≥ 1 mg/kg methylprednisolone or equivalent) within 100 days post-transplant. Among these variables, only the use of ≥ 1 mg/kg methylprednisolone within 100 days was significantly associated with breakthrough CMV DNAemia (*p* = 0.016). No statistically significant associations were observed for the other factors examined.

**Table 2:** Univariate analysis of variables associated with breakthrough CMV DNAemia.

Variables	Non-breakthrough (*N* = 60)	Breakthrough(*N* = 14)	*P*	HR(95%CI)
Patient-related variables				
Age(years), mean ± SD	37.38 ± 15.13	33.29 ± 13.62	0.353	0.941–1.022
Sex, n (%)	33(55%)	11(78.5%)	0.117	0.084–1.317
Donor-related variables				
Age(years), mean ± SD	32.17 ± 12.38	33.86 ± 9.19	0.628	0.963–1.064
Sex, n (%)	48(80.0%)	13(92.8%)	0.278	0.037–2.589
Transplant-related variables				
Gender consistency between patient and donor, n (%)	31(51.7%)	10(71.4%)	0.188	0.660–8.288
HLA Matched related donor, n (%)	5(8.3%)	1(7.1%)	0.883	0.127–10.996
Diagnosed as aplastic anemia, n (%)	9(15.0%)	1(7.1%)	0.450	0.051–3.757
Use of ≥1 mg/kg methylprednisolone within 100 days or its equivalent, n (%)	8(13.3%)	6(42.8%)	0.016	1.337–17.781

1. Values are presented as mean ± SD or n (%), as appropriate.

HLA: Human Leukocyte Antigen. SD: Standard Deviation. HR: Hazard Ratio.

### Clinical outcomes observed during letermovir prophylaxis and follow‑up

At a median follow-up of 17.73 months, the mean OS was 17.26 ± 0.72 months (95%CI, 15.84–18.67), and the mean PFS was 16.21 ± 0.84 months (95%CI, 14.56–17.86). EBV DNAemia was detected in 31.08% (23/74) of patients, and BKPyV-HC occurred in 16.22% (12/74). The median peak EBV viral load was 2,100.00 copies/mL (IQR, 6,200 copies/mL). PTLD was observed in 5.41% (4/74) of patients. All PTLD cases were of B-cell origin, including monomorphic and polymorphic subtypes.

## Discussion

In this real-world study, we described the incidence and clinical characteristics of CMV DNAemia in Chinese allo-HSCT recipients receiving letermovir prophylaxis under the Beijing Protocol. Breakthrough CMV DNAemia was observed in a proportion of patients during prophylaxis, whereas CMV end-organ disease was uncommon. In addition, descriptive analyses identified several clinical and pharmacokinetic characteristics observed among patients with breakthrough CMV DNAemia. The incidence of CMV DNAemia varies widely across HSCT types, ranging from 18% to 85%, with a median onset of 32–41 days post-transplant ^2,3,23–26^. In our cohort, sensitivity analyses excluding the small number of D−/R+ cases suggested minimal influence on the primary findings. Given the retrospective single‑arm design and absence of a comparator group, this study was not intended to evaluate prophylactic efficacy, and comparisons with previously published studies are provided solely for clinical context.

The observed time‑dependent associations between CMV DNAemia and aGVHD are biologically plausible and consistent with established post‑transplant pathophysiology. CMV reactivation has been shown to induce immune dysregulation and pro‑inflammatory responses that may contribute to the development of aGVHD. Conversely, treatment of aGVHD, particularly intensified immunosuppressive therapy, can impair antiviral immune surveillance and predispose patients to CMV reactivation ^27^. Such bidirectional interactions between CMV replication and aGVHD have been described in prior clinical and experimental studies ^28,29^, supporting a close temporal interplay. In addition, several real-world studies in the systematic review ^30^ from Western countries have reported a lower incidence of aGVHD among patients receiving letermovir prophylaxis. In our cohort, the overall incidence of aGVHD was also relatively low (17.57%). However, this observation should be interpreted cautiously. The absence of a contemporaneous control group and temporal changes in transplant practices, including donor type, conditioning intensity, and immunosuppressive regimens ^31^, preclude any causal inference regarding the association between letermovir prophylaxis and aGVHD risk in this observational study. Accordingly, this finding should be considered hypothesis-generating, and further prospective studies are warranted to clarify this relationship.

Several studies ^14,28,29^ have explored clinical and treatment‑related characteristics observed in patients with breakthrough CMV DNAemia during letermovir prophylaxis, including transplant characteristics and drug exposure. Some studies ^14,32,33^ have noted that lower letermovir Cmin (‹ 2731 ng/mL) may be more frequently observed among patients with CMV DNAemia. In addition ^34^, delayed initiation of letermovir prophylaxis has been described in association with CMV reactivation ^34^, and the 10th European Conference on Infections in Leukaemia guideline ^35^ therefore recommends early initiation of prophylaxis when feasible. In the present study, patients with breakthrough CMV DNAemia initiated letermovir significantly later than those without breakthrough CMV DNAemia, with a mean time to initiation of 19 days post‑transplant. This delay may reflect real‑world factors, including economic considerations, reimbursement policies, and patient willingness, which can influence the timing of prophylaxis initiation in routine clinical practice.

Furthermore, data from the national registry database of the Japanese Society for Transplantation and Cellular Therapy ^36^ have highlighted transplant‑related characteristics, such as cord blood transplantation, use of ATG or PTCY, and grade II–IV acute GVHD, as being frequently observed in patients with clinically significant CMV infection.

Consistent with these findings, our descriptive analyses showed that lower letermovir C_min_, delayed initiation of prophylaxis, and administration of methylprednisolone at doses≥ 1 mg/kg within 100 days post-transplant were more commonly observed among patients with breakthrough CMV DNAemia. These factors likely reflect the combined effects of antiviral exposure and impaired immune control in the early post‑transplant period. Given the observational design, limited sample size, and complexity of allo‑HSCT management, these observations should be considered hypothesis‑generating, and further prospective studies are warranted.

Secondary prophylaxis has been increasingly used in allo‑HCT recipients with breakthrough CMV DNAemia after primary prophylaxis or preemptive therapy. In the present study, no recurrent CMV DNAemia was observed during the observation period in this subgroup. Similar low recurrence rates have been described in prior reports, although these findings should be interpreted cautiously given differences in patient populations and study designs ^37–39^ Patients receiving secondary prophylaxis are generally at high risk for CMV reactivation, including exposure to ATG, HLA‑mismatched or haploidentical donors, aGVHD, and corticosteroid‑based immunosuppression ^38^. Consistent with this clinical context, all eight patients in our study underwent haploidentical transplantation with ATG, and five required high‑dose corticosteroids for aGVHD.

The optimal duration of secondary prophylaxis remains uncertain. In this study, a mean duration of 52 ± 28 days, which was shorter than previously reported ^37–39^. No recurrent CMV DNAemia was observed during the follow‑up. However, given the small sample size and observational design, these findings should be regarded as descriptive and hypothesis‑generating. Further prospective studies are needed to clarify the appropriate duration of secondary prophylaxis and to explore how immunosuppressive burden, GVHD activity, immune reconstitution, and antiviral exposure may interact to influence CMV outcomes.

A phase III randomized trial ^40^ reported that discontinuation of letermovir prophylaxis at approximately day 100 post‑HSCT was followed by an increased incidence of csCMVi between days 100 and 200, with a cumulative incidence of 12.1%. Similarly, several single‑center retrospective studies ^41–44^ reported clinically significant CMV infection (csCMVi) rates ranging from approximately 10% to over 20% after stopping letermovir at day 100. These findings provide important contextual information regarding the timing of CMV events following prophylaxis discontinuation. In the present single‑arm study, CMV viremia during follow‑up was observed in 8.45% of patients. Nearly half of the patients received letermovir prophylaxis beyond 100 days, reflecting substantial heterogeneity in real‑world prophylaxis duration. A recent retrospective study ^45^ reported differences in csCMVi incidence at day 200 between shorter and longer durations of letermovir prophylaxis, whereas the cumulative incidence appeared comparable by day 400, highlighting the complexity of interpreting duration‑dependent effects across studies. Collectively, prior studies have raised the hypothesis that CMV reactivation after prophylaxis discontinuation may be associated with the pace and quality of immune reconstitution. In this context, CMV‑specific cell‑mediated immunity (CMV‑CMI)–guided letermovir prophylaxis has gained increasing attention, with emerging evidence supporting individualized prophylaxis strategies based on host immune recovery ^46.^ However, these approaches remain investigational, and prospective studies are required to define the optimal duration and monitoring strategies for letermovir prophylaxis in high‑risk patients.

Concerns have been raised regarding the occurrence of other viral infections during letermovir prophylaxis. In previous studies, the reported incidence of EBV DNAemia has ranged from 8.8% to 58.8% ^47–49^. While EBV-PTLD remains relatively uncommon^2,50–52^. In the present study, the incidences of EBV DNAemia and PTLD were within the range in the literature. It should be noted that

most Chinese allo-HSCT recipients are EBV IgG–positive ^42^, frequently undergo haploidentical or HLA-mismatched transplantation, and commonly receive high-dose ATG (10 mg/kg) for GVHD prophylaxis—factors that are well recognized to impair immune reconstitution and increase the risk of EBV reactivation and PTLD ^53^. Given that nearly all patients in this cohort were exposed to high‑dose ATG, the independent contribution of letermovir prophylaxis to EBV‑related events could not be assessed, and any potential association should therefore be interpreted with caution.

BK polyomavirus–associated hemorrhagic cystitis remains a clinically relevant complication after allo‑HSCT ^54–56^. Although CMV DNAemia has been reported as a risk factor for BKPyV‑HC in prior studies ^57,58^, the present study was not designed to evaluate potential interactions among CMV, BKPyV, and antiviral prophylaxis, and no causal inferences can be drawn. Further studies are required to clarify the complex relationships among viral reactivation, immune reconstitution, and prophylactic strategies in this setting ^59^.

This study has several limitations. First, the retrospective, single‑arm design may introduce selection bias and reflect center‑specific clinical practices, thereby limiting the generalizability of the findings. Second, the relatively small sample size reduces statistical power and may limit the robustness of exploratory analyses, particularly for subgroup evaluations. In particular, the small number of D₊ / R₋ cases limited subgroup inference, although sensitivity analyses suggested no material impact on the overall estimates. Third, the absence of a contemporaneous control group without letermovir prophylaxis precludes direct assessment of prophylactic efficacy and represents a major limitation of this study. In addition, the retrospective design and reliance primarily on univariate analyses restrict the ability to fully control for potential confounders and preclude causal inference. Fourth, mechanistic insights were limited, as letermovir resistance testing and systematic pharmacokinetic monitoring were not routinely performed, and detailed immune reconstitution assessments were unavailable. Moreover, the near‑universal use of high‑dose ATG in this cohort may have confounded analyses of other viral infections and limited the ability to disentangle the independent effects of different immunosuppressive components. Finally, although letermovir exposure may be influenced by concomitant medications, formal adjustment for drug-drug interactions was not performed. While prophylactic and immunosuppressive regimens were relatively homogeneous within this center, residual confounding related to concomitant therapies and real‑world treatment complexity cannot be fully excluded.

In conclusion, this real-world study describes the occurrence of CMV DNAemia in allo-HSCT recipients receiving letermovir prophylaxis under the Beijing Protocol. Observed differences in immunosuppressive exposure, letermovir exposure, and timing of prophylaxis initiation highlight areas that warrant further investigation. Prospective studies are needed to inform optimized and individualized CMV prophylaxis strategies in high‑risk transplant recipients.

## Supplementary Information

Below is the link to the electronic supplementary material.

Supplementary Material 1

## Data Availability

The datasets were obtained from existing clinical records. The data are publicly available through the Chinese Clinical Trial Registry (https://www.chictr.org.cn/index.html) under the registration number ChiCTR2500100795. Additional information may be obtained from the corresponding author upon reasonable.
