# Supplementary material for: Letermovir prophylaxis and early CMV DNAemia after allogeneic hematopoietic stem cell transplantation: a real‑world study from China
Source: Sci Rep. 2026 Apr 29;16:19969. doi: 10.1038/s41598-026-50851-1 (PMC13319509; doi:10.1038/s41598-026-50851-1)
Supplement: Supplementary file 1 — Supplementary Material 1 [file 41598_2026_50851_MOESM1_ESM.docx]

**Table 1: Transplantation regimen of 74 patients**

| Conditioning | | Graft Source | | GVHD prophylaxis | |
| --- | --- | --- | --- | --- | --- |
| Relapsed or refractory ALL | Total Body Irradiation/Thiotepa or Cyclophosphamide | **Aplastic anemia (donor and recipient blood type consistency)** | Bone marrow plus peripheral blood stem cells | **Basic regimen (all enrolled participants)** | Cyclosporin A plus Mycophenolate mofetil plus short-term Methotrexate |
| Central nervous system leukemia, underlying heart disease, salvage transplantation. | Thiotepa/Busulfan/Fludarabine or Mitoxantrone Hydrochloride Liposome | **Aplastic anemia (donor-recipient blood type inconsistency); Severe myelodysplasia,**  **highly malignant conditions** | Umbilical cord blood (Shandong Umbilical Cord Blood Bank) plus peripheral blood stem cells | **HLA haploidentical matched unrelated donor HSCT** | Basic regimen plus Anti-Human Thymocyte immunoglobulin (ATG; 10 mg/kg) administered during conditioning |
| Aplastic anemia | Fludarabine/Anti-Human Thymocyte immunoglobulin/Cyclophosphamide or Busulfan | **Others** | Peripheral blood stem cells | **HLA identical related donor, with female donors aged ＞40 years or with prior childbirth history** | Basic regimen+ Anti-Human Thymocyte immunoglobulin (5 mg/kg) administered during conditioning |
| Others | Modified Cytarabine/Busulfan/Cyclophosphamide or Semustine |  |  | **CD3^+^ cells count ≥4×10^8^/kg from donor peripheral blood stem cells** | Basic regimen plus two doses of basiliximab administered on days +1 and +4 |
